# Population distribution and burden of acute gastrointestinal illness in British Columbia, Canada

**DOI:** 10.1186/1471-2458-6-307

**Published:** 2006-12-19

**Authors:** M Kate Thomas, Shannon E Majowicz, Laura MacDougall, Paul N Sockett, Suzie J Kovacs, Murray Fyfe, Victoria L Edge, Kathryn Doré, James A Flint, Spencer Henson, Andria Q Jones

**Affiliations:** 1 Foodborne, Waterborne and Zoonotic Infections Division, Public Health Agency of Canada, Guelph and Ottawa, Ontario, Canada; 2 Department of Population Medicine, University of Guelph, Guelph, Ontario, Canada; 3 British Columbia Centre for Disease Control and Prevention, Vancouver, British Columbia, Canada; 4 Western University of Health Sciences, Pomona, California, USA; 5 Vancouver Island Health Authority, Victoria, British Columbia, Canada; 6 Department of Agricultural Economics and Business, University of Guelph, Guelph, Ontario, Canada; 7 Division of Community Health, Faculty of Medicine, Memorial University of Newfoundland, St. John's, Newfoundland and Labrador, Canada

## Abstract

**Background:**

In developed countries, gastrointestinal illness (GI) is typically mild and self-limiting, however, it has considerable economic impact due to high morbidity.

**Methods:**

The magnitude and distribution of acute GI in British Columbia (BC), Canada was evaluated via a cross-sectional telephone survey of 4,612 randomly selected residents, conducted from June 2002 to June 2003. Respondents were asked if they had experienced vomiting or diarrhoea in the 28 days prior to the interview.

**Results:**

A response rate of 44.3% was achieved. A monthly prevalence of 9.2% (95%CI 8.4 – 10.0), an incidence rate of 1.3 (95% CI 1.1–1.4) episodes of acute GI per person-year, and an average probability that an individual developed illness in the year of 71.6% (95% CI 68.0–74.8), weighted by population size were observed. The average duration of illness was 3.7 days, translating into 19.2 million days annually of acute GI in BC.

**Conclusion:**

The results corroborate those from previous Canadian and international studies, highlighting the substantial burden of acute GI.

## Background

Gastrointestinal illness (GI) is a global public health concern. In developed countries, GI is typically mild and self-limiting, but has considerable economic impact due to high morbidity [1-3]. Recent studies on the burden of GI in the general population of a number of countries have been reported [4-12]. To estimate the burden of GI in the Canadian population, the Public Health Agency of Canada (PHAC; formerly Health Canada) developed the National Studies on Acute Gastrointestinal Illness (NSAGI) initiative in 2000. Population-based studies, designed to describe self-reported, acute GI in selected Canadian populations, are part of this initiative. In March 2002, the PHAC completed the first such population study in the City of Hamilton, Ontario, Canada [13]. In order to determine if the burden of GI was the same across the country, a second population study was completed in the province of British Columbia (BC) in June 2003. Additionally, since public health in Canada is primarily a provincial responsibility, this study was conducted to provide information to BC policy makers. The current paper describes the frequency, magnitude, distribution and clinical burden of acute, self-reported GI in BC.

## Methods

### Study design

A cross-sectional telephone survey was administered from June 2002 to June 2003 to randomly selected residents within a defined study area in BC. The study area consisted of a convenience sample of three local public health authority regions, which were chosen to approximate different community types of the BC population. Taken together, these three areas are similar to the overall BC population with respect to age and gender, and represent approximately 19% of the BC population (4 million people; [14]). The areas chosen were, one urban region (Vancouver; population 550,000), one rural region (East Kootenay; population 80,000), and one semi-urban and rural region (Northern Interior; population 132,000).

Survey methods were consistent with the previous Canadian population study to ensure comparability [13]. The sampling frame consisted of a randomized list of residential telephone numbers obtained from a commercial database (SelectPhone™, InfoUSA, Inc.). All households in the sampling frame with mailing addresses were sent an introductory letter one to two weeks prior to the first telephone call attempt, in order to increase the response rate [15]. The letter explained the purpose of the study, how individuals had been selected to participate and the necessity of random selection. Initial contact with a member of each household was attempted up to five times on different days and at different times of the day. One individual from each household was then randomly selected to participate by identifying the household member who had the next birthday. Up to five attempts were made to contact the selected individual to complete the survey. Respondents were informed that the survey was optional, confidential and anonymous and their verbal consent was obtained. Surveys were administered in English, French or Cantonese, as requested by the respondent.

A target sample size of 1,536 interviews per region was calculated to detect a prevalence of 10%, with a 5% level of precision and a 1.5% allowable error. This translated into a total of 4,608 interviews, with an overall allowable error of 0.76% (EpiInfo Version 6.04). To achieve this target, approximately 128 interviews were completed per region per month, for 12 months.

The survey was developed by modifying a previous Canadian population survey. To be consistent with other previously conducted studies, respondents were asked if they had experienced any vomiting or diarrhoea in the 28 days prior to the interview. Cases of acute, self-reported GI were those who had experienced any diarrhoea or vomiting in the 28 days prior to the interview, where diarrhoea was defined as any loose stool or stool with abnormal liquidity. A broad case definition was deliberately chosen to ensure high sensitivity. Respondents who identified more than one episode of GI, separated by seven days or more, during the 28 days prior to the interview, were asked to respond only for their most recent episode. Respondents who did not report symptoms of GI, as well as those identified as having chronic GI, were included in the non-case category as in the previous Canadian population study [13]. Chronic GI was identified by statements indicating that symptoms resulted from pregnancy, medication use, food allergy and/or medical condition previously diagnosed by a doctor (e.g. colitis, diverticulitis, Crohn's disease, irritable bowel syndrome).

Additional survey questions explored secondary symptoms, medical history, severity of illness, loss of productivity, daycare attendance, occupation, health care and medication use, international travel, regional location, demographic characteristics, drinking water consumption, and illness in other members of the household. Only the results from questions relating to the objectives of this paper are reported here.

### Ethical approval

Ethical approval to conduct this study was obtained from the Human Subjects Committee of the University of Guelph (Guelph, Ontario, Canada), and the University of British Columbia Behavioural Research Ethics Board (Vancouver, BC, Canada).

### Data analysis

Data were analysed using Microsoft Excel 2000 (Microsoft Corporation, Redmond, WA, USA) and SAS version 8.01 (SAS Institute Inc., Cary, NC, USA). Individuals responding 'don't know/unsure' or that refused to answer any question were excluded from the analysis of that question. For incidence rate calculations, respondents identifying multiple episodes were counted as a single episode. The primary outcome measure of monthly prevalence was defined as the number of respondents reporting acute GI in the previous 28 days divided by the total number of respondents. Prevalence, incidence rate and incidence proportion calculations were performed using formulas obtained from Modern Epidemiology (Appendix 1; [16]).

Differences between medians were tested the Median Test. The null hypothesis of no overall association between the prevalence of disease and each demographic factor (age group, cultural group, gender, total household income level, household size, region and the highest level of education attained) was tested using the Mantel-Haenszel χ^2 ^Test. Within each demographic factor, the difference between the proportion of cases (i.e. the prevalence of GI) at a given level of the factor and the proportion of cases at all other levels of that factor combined was tested using the χ^2^Test.

Multivariate analysis of these variables and their interaction terms was done using manual forward, backward stepwise and best subsets logistic regression, with the 95% confidence interval being used to determine a variable's significance. Variables with a p-value less than 0.10 were included in the final model.

## Results

### Response rate and magnitude of illness

**Figure 1:**
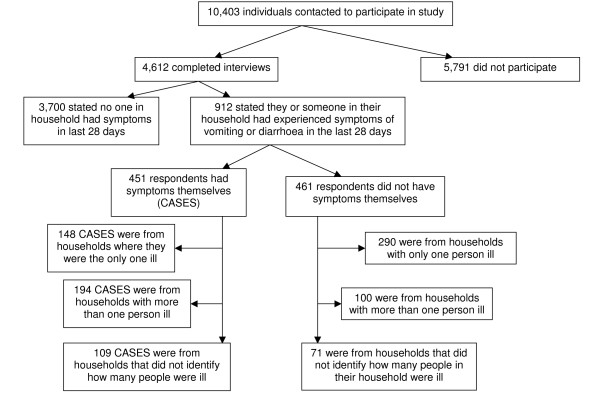
Response rate and survey outcomes for the retrospective telephone survey of acute gastrointestinal illness conducted in the province of British Columbia, Canada.

Complete interviews were conducted with 4,612 of the 10,403 individuals contacted to participate in this study, yielding an overall response rate of 44.3% (Figure 1). Northern Interior was the region with the highest response rate (47%), followed by East Kootenay (45%) and Vancouver (41%). Of the 4,612 respondents, 912 (19.8%; 95% CI 18.6–20.9) reported that they or someone in their household had experienced symptoms of vomiting or diarrhoea in the previous 28 days. This included 682 respondents who reported that they themselves experienced symptoms of vomiting or diarrhoea in the previous 28 days, however 131 (2.8%; 95% CI 2.4 – 3.3) were identified as chronic cases and included in the non-case category, leaving 451 respondents (9.8%; 95% CI 8.9–10.6) to be identified as cases. Of those 451 cases, 144 (31.9%) reported more than one episode during the 28 days prior to the interview.

Prevalence varied by region, being lowest in Vancouver (8.7%), followed by East Kootenay (9.6%) and the Interior region (11.1%); this difference was statistically significant (p = 0.029). The overall study prevalence, weighted by population size, was 9.2% (95% CI 8.4 – 10.0). The incidence rate for the population, weighted by population size, was 1.3 (95% CI 1.1–1.4) episodes per person-year, and the annual incidence proportion, weighted by population size, was 71.6% (95% CI 68.0 – 74.8). A total of 58 people had symptoms of diarrhoea or vomiting on the day of interview, yielding a point prevalence of 1.3% (95% CI 0.9 – 1.6).

### Distribution

**Figure 2:**
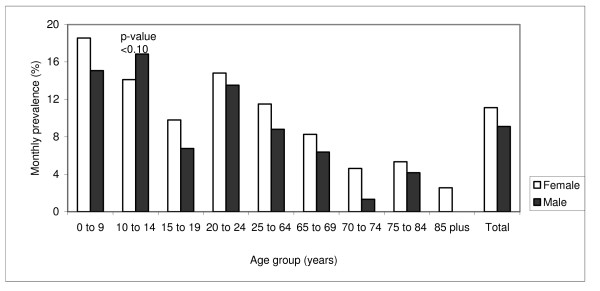
Monthly prevalence of acute gastrointestinal illness by age group and gender, in the province of British Columbia, Canada, showing p-values < 0.10, indicating association between prevalence and gender, per age group.

**Figure 3:**
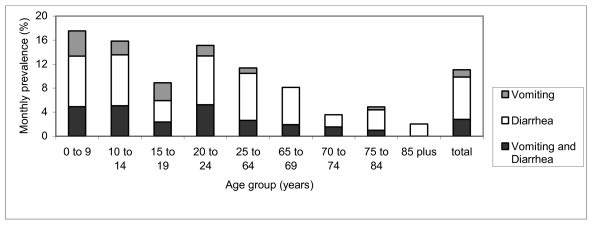
Monthly prevalence of acute gastrointestinal illness by age group and primary symptom, in the province of British Columbia, Canada.

The monthly prevalence of illness per demographic category is shown (Table 1). There were significantly more female cases than male cases (p = 0.018). Cases were significantly younger than non-cases (median age of 40 and 46 years, respectively; p < 0.001). Male respondents were significantly younger than female respondents (median age of 43 and 47 years, respectively; p < 0.001). Prevalence by age and gender is shown (Figure 2). Of the 451 cases, 51 (11.3%) experienced only vomiting, 286 (63.4%) experienced only diarrhoea, and 114 (25.3%) experienced diarrhoea and vomiting. The age distribution by primary symptom is shown (Figure 3).

### Univariate analysis

**Figure 4:**
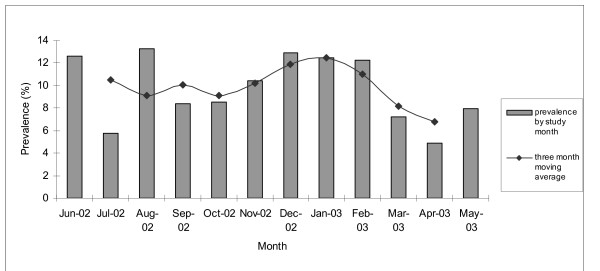
Monthly prevalence of acute gastrointestinal illness by study month with three month moving average, in the province of British Columbia, Canada.

**Figure 5:**
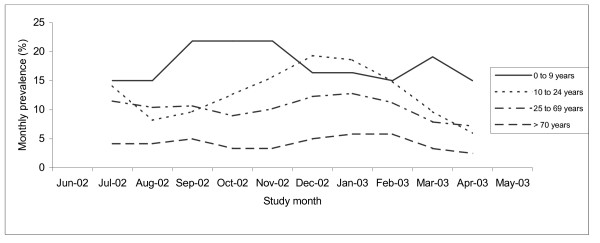
Three-month moving average of monthly prevalence of acute gastrointestinal illness by study month and age group (in years), in the province of British Columbia, Canada.

Illness was significantly associated with level of education (p = 0.008), number of people in household (p < 0.001), age group (p < 0.001) and gender (p = 0.018), but not with total household income (p = 0.271) or cultural group (p = 0.125). There was variation in the prevalence of GI throughout the year, peaking in August (13.3%) and December (12.9%; Figure 4). Figure 5 illustrates the temporal distribution of GI cases by age group. No real seasonal pattern in the prevalence of GI was observed in those 25 years of age and older, however the prevalence in those 10–24 years of age peaked in July and December. The prevalence in those 0–9 years of age followed a different temporal pattern, with a 'peak' in the fall (September, October, November) and a second peak in the spring (March).

### Multivariate analysis

Multivariate analysis of the prevalence of GI was undertaken with age group, gender, region, total household income, highest level of education attained, household size, and cultural group as explanatory variables. Variables with a p-value < 0.10 were kept in the model. The significant variables in the final model were age group (p-value < 0.001) and gender (p-value = 0.02; Table 2). Compared to the referent group of those 25–64 years of age, and adjusting for gender, those 0–9 and 10–14 years of age were 1.8 and 1.6 times more likely to experience GI, respectively, and those 70–74 years of age were 3.3 times less likely. Females were 1.3 times more likely to experience GI than males, adjusting for age.

### Symptoms and severity

Of 400 respondents with diarrhoea, 21 (5.3%) had bloody diarrhoea: 12 (3%) reported 'just a little blood in the toilet or on the toilet paper'; 8 (2%) reported 'some blood mixed with stool'; 1 (0.3%) reported 'so much blood that the stool was almost entirely blood'. The frequency of secondary symptoms is shown (Table 3). On their worst day of symptoms, cases reported vomiting an average of three times (range 1–15 times), and an average of four loose stools (range of 1–20). Restricted activity was reported by 234 (51.9%) cases. Of these, cases indicated that they were hospitalized (4; 1.7%), or spent time home confined to bed (114; 48.7%), or spent time at home unable to do most of their normal activities (121; 51.7%). On average the duration of illness was 3.7 days.

### Predisposing factors

Cases were asked about their use of medications with possible gastrointestinal effects that were taken in the 28 days prior to their illness. In total 113 cases took medications; specifically 87 (19.3%) cases had taken antacids, 25 (5.5%) antibiotics, 6 (1.3%) laxatives, and 3 (0.7%) immunosuppressive agents in the four weeks prior to becoming ill.

### Health care seeking behaviour

Sixty-two (13.8%) cases telephoned a health care provider one or more times and 52 cases (11.6%) visited a health care provider a total of 59 times for their illness (Table 4). For those visiting a doctor, the number and type (blood, diarrhoea, urine, or vomit) of samples requested by the physician, as well as compliance with these requests, are shown (Table 5). Of the 52 cases that visited a health care provider, 28 cases were asked to submit a total of 50 samples; 42 samples were submitted. Six of the ten cases who submitted a stool sample knew the result of the laboratory test: five were negative; one was positive for *Clostridium*.

To self-treat their illness, 288 (63.9%) cases used one or more over the counter medications: 190 (66.0%) used painkillers, 80 (27.8%) used anti-diarrhoeals, 60 (20.8%) used anti-nauseants and 59 (20.5%) used herbal remedies.

### Impact on work and school

Of the 212 respondents who had GI aged 18 years and older, and who were employed, 69 (32.5%) took time away from work because of their illness, 30 (43.5%) lost personal income as a result, but none lost their job due to illness. The median and mean numbers of days away from work as a result of GI were 2.0 and 2.4, respectively, (range: 1.5 hours to 12 days). Four of the eleven children normally attending daycare were unable to attend as a result of their illness (range: 1 to 3 days missed). Eight parents with children aged 15 years or less took time away from work due to their children's illness. Twenty-seven (36.0%) of the 75 children, (less than 18 years) and enrolled in school, missed school as a result of their illness. The median and mean numbers of days of school missed were 2.0 and 1.9, respectively (range: 0.5 to 5 days).

## Discussion

The results reported here match those from the previous Canadian study [13], where the incidence rate was 1.3 episodes per person-year, the monthly prevalence was 10%, the average annual probability was 71% and the point prevalence was 1.46%, confirming that the magnitude of gastrointestinal illness at the community level in Canada is considerable and does not appear to vary with geography. These results are also similar to those reported in recent international studies on acute GI in developed countries [7,9,12,17]. However, due to 'telescoping', estimates based on retrospective studies may overestimate results compared to those generated by prospective studies. The study in England found that their retrospective estimate was nearly three times that of their prospective estimate [17]. Likely the point prevalence is a better measure of prevalence as it is less subject to 'telescoping'.

Because the three study regions capture the full spectrum of community types in BC, the results of this study can be reasonably extrapolated to the entire province. In doing so, we estimate roughly 400,000 cases of acute GI per month in BC, with each individual experiencing an estimated 4.8 person-days of acute GI annually. This would translate into 19.2 million days of acute GI experienced in BC each year. Additionally, we would expect over 12 million missed workdays by adults, and over 10 million missed school days among children in BC each year as a result of acute GI. Thus the burden of acute GI in the BC population remains substantial and is associated with discomfort, disability and a significant number of days of lost productivity due to sick time or time taken to care for relatives. In contrast chronic disease surveillance in 2001 identified 574 deaths per 100,000 BC residents per year, though direct comparison with the calculated burden of GI presented here is impossible, this does present a crude value for relative comparison [18].

Much discussion has occurred around comparing results of studies that have used different case definitions. Currently there is a group of international researchers attempting to resolve this issue [19,20] This study used a broad case definition for high sensitivity and case capture. Other studies have used more strict definitions for diarrhoea [6,7,9,21-23]. The change resulting from redefining our definition of diarrhoea as three or more loose stools or stools with abnormal liquidity in 24 hours is minimal: the average monthly prevalence would be 8.8% (95% CI 8.0 – 9.6), the average annual probability would be 69.8% (95% CI 66.2 – 73.3) and the incidence rate would be 1.1 (95% CI 1.0 – 1.3) episodes of acute GI per person-year. Even under this stricter definition, the burden remains substantial at over 17 million days of acute GI experienced in BC each year. Further research into the impact of choice of symptomatic case definition is needed, and ultimately a validated, standard definition would be used to ensure comparability between studies.

The age and gender distribution of acute GI in this study is similar to other reported results[7]. Significantly more female than male cases were observed. Females in the 25–64 year old age group had a slightly higher prevalence though not statistically significant; in the previous Canadian study, this difference was significant [13]. Studies in England [24] and Ireland [9] also reported an increase in GI among females in the 25–44 year old age group. Higher rates in this adult age group may represent increased exposure of parents with children [25], and for women in particular, this may also be the result of relatively more exposure in the kitchen during meal preparation (e.g. handling of raw products) [26].

The temporal distribution of acute GI for individuals in the 10–24 year old age group followed a similar pattern to other studies, with a bimodal distribution peaking in the summer and winter months [6,13]. This probably reflects the association of bacterial and parasitic gastrointestinal diseases with a summer seasonality [27,28], and viral gastrointestinal disease with a winter seasonality [29]. However, those in the 0–9 year old age group had a different temporal distribution, reporting peaks in the fall and spring months. This is consistent with rotavirus and norovirus infections, which are common in young children and have been shown to have peaks occurring from, fall to spring [30,31]. In the previous Canadian study [13] there was no temporal trend for this age group, and this is likely due to that study's smaller sample size.

A limitation of this study was its low response rate (44.3%); although it is consistent with recent similar international studies [6,9,12], and is higher than that in the previous Canadian study (36.6%; [13]), potentially due in part to the introductory letter [15]. A potential bias associated with low response is non-response bias, which has been discussed in detail elsewhere [13].

This study was administered by telephone and thus will not capture those who do not own a telephone (1.4% of BC residences do not own at least one telephone or cellular telephone number [32]), or those who may not be home due to their illness. There is the potential that the rate of illness for those without telephones is different to the rate of illness of those with telephones and thus these results could be biased. For example, if not having a telephone is related to lower income and higher disease rate, then this may underestimate the incidence and prevalence of acute GI in the population, however if not having a telephone is associated with higher income- as may be the case given the emergence of cellular phones- and lower disease rate, this may overestimate the incidence and prevalence of acute GI in the population. As well if individuals were in hospital or institutionalized or had died as a result of their illness, this would not been captured and would thus potentially bias the results, by underestimating the incidence, prevalence and severity of acute GI in the population.

## Conclusion

This study illustrates that acute gastrointestinal illness (GI) is a significant burden in the British Columbian population, with higher rates in women and those under 15 years of age, and confirms that the burden of GI is similar between Canadian populations over varying geography. Additionally the burden of acute GI in Canada is similar to other developed countries. Future research activities in Canada should identify the costs and actual causes associated with acute GI in the population in order to better design intervention strategies. Looking internationally, a standardized case definition would be useful to determine the global burden of acute GI and for comparison between countries and studies.

## Appendix 1

Formulas for calculating prevalence, incidence rate and incidence proportion (16).

Prevalence:

=#ofcasesTotal#atrisk
 MathType@MTEF@5@5@+=feaafiart1ev1aaatCvAUfKttLearuWrP9MDH5MBPbIqV92AaeXatLxBI9gBaebbnrfifHhDYfgasaacH8akY=wiFfYdH8Gipec8Eeeu0xXdbba9frFj0=OqFfea0dXdd9vqai=hGuQ8kuc9pgc9s8qqaq=dirpe0xb9q8qiLsFr0=vr0=vr0dc8meaabaqaciaacaGaaeqabaqabeGadaaakeaacqGH9aqpdaWcaaqaaiabcocaJiabd+gaVjabdAgaMjabdogaJjabdggaHjabdohaZjabdwgaLjabdohaZbqaaiabdsfaujabd+gaVjabdsha0jabdggaHjabdYgaSjabcocaJiabdggaHjabdsha0jabdkhaYjabdMgaPjabdohaZjabdUgaRbaaaaa@47EC@

Annual incidence rate:

=#ofcases12[(Total#atrisk)+(Total#atrisk−#ofcases)]×36528
 MathType@MTEF@5@5@+=feaafiart1ev1aaatCvAUfKttLearuWrP9MDH5MBPbIqV92AaeXatLxBI9gBaebbnrfifHhDYfgasaacH8akY=wiFfYdH8Gipec8Eeeu0xXdbba9frFj0=OqFfea0dXdd9vqai=hGuQ8kuc9pgc9s8qqaq=dirpe0xb9q8qiLsFr0=vr0=vr0dc8meaabaqaciaacaGaaeqabaqabeGadaaakeaacqGH9aqpfaqaaeGabqaabaGaei4iamIaem4Ba8MaemOzayMaem4yamMaemyyaeMaem4CamNaemyzauMaem4CamhabaWaaSGaaeaacqaIXaqmaeaacqaIYaGmaaGaei4waSLaeiikaGIaemivaqLaem4Ba8MaemiDaqNaemyyaeMaemiBaWMaei4iamIaemyyaeMaemiDaqNaemOCaiNaemyAaKMaem4CamNaem4AaSMaeiykaKIaey4kaSIaeiikaGIaemivaqLaem4Ba8MaemiDaqNaemyyaeMaemiBaWMaei4iamIaemyyaeMaemiDaqNaemOCaiNaemyAaKMaem4CamNaem4AaSMaeyOeI0Iaei4iamIaem4Ba8MaemOzayMaem4yamMaemyyaeMaem4CamNaemyzauMaem4CamNaeiykaKIaeiyxa0faaiabgEna0oaalaaabaGaeG4mamJaeGOnayJaeGynaudabaGaeGOmaiJaeGioaGdaaaaa@72BA@

Annual incidence proportion:

= 1 - ⌊(1 - *x*)^365/28^⌋

where x=#ofcasesTotal#atrisk−12#ofwithdrawals
 MathType@MTEF@5@5@+=feaafiart1ev1aaatCvAUfKttLearuWrP9MDH5MBPbIqV92AaeXatLxBI9gBaebbnrfifHhDYfgasaacH8akY=wiFfYdH8Gipec8Eeeu0xXdbba9frFj0=OqFfea0dXdd9vqai=hGuQ8kuc9pgc9s8qqaq=dirpe0xb9q8qiLsFr0=vr0=vr0dc8meaabaqaciaacaGaaeqabaqabeGadaaakeaacqqG3bWDcqqGObaAcqqGLbqzcqqGYbGCcqqGLbqzcqqGGaaicqqG4baEcqGH9aqpfaqaaeGabqaabaGaei4iamIaem4Ba8MaemOzayMaem4yamMaemyyaeMaem4CamNaemyzauMaem4CamhabaGaemivaqLaem4Ba8MaemiDaqNaemyyaeMaemiBaWMaei4iamIaemyyaeMaemiDaqNaemOCaiNaemyAaKMaem4CamNaem4AaSMaeyOeI0YaaSGaaeaacqaIXaqmaeaacqaIYaGmaaGaei4iamIaem4Ba8MaemOzayMaem4DaCNaemyAaKMaemiDaqNaemiAaGMaemizaqMaemOCaiNaemyyaeMaem4DaCNaemyyaeMaemiBaWMaem4Camhaaaaa@66A5@

Values:

# of cases = 451

Total # at risk = 4612

# of withdrawals = 0

## Competing interests

The author(s) declare that they have no competing interests.

## Authors' contributions

KT performed analysis and data interpretation as well as drafted and revised the manuscript. SM was part of the research team that designed the National Studies of Acute Gastrointestinal Illness (NSAGI), assisted with data interpretation and provided comment and revisions for the manuscript. LM assisted in the design of the BC NSAGI project, assisted with data interpretation and manuscript revisions. PS contributed substantially to the original conception of the NSAGI studies and the design of this specific study, reviewed the data, analysis and interpretation and provided critical review of this manuscript. SK participated in the data cleaning and analysis and critically reviewed the manuscript. MF participated in the conception of this study, interpretation of the analysis and critically reviewed this manuscript. VL contributed to the conception of the study, the interpretation of the data and analysis, and provided comment and review of the manuscript. KD was part of the research team that designed NSAGI and provided critical review of the manuscript. JF was part of the research team that designed NSAGI, development of the study methodology and provided critical review of the manuscript. SH assisted in conception of the study, interpretation of data and provided critical review of the manuscript. AJ assisted with interpretation of the data and provided critical review of the manuscript. All authors read and give final approval of the final manuscript for publication.

## Pre-publication history

The pre-publication history for this paper can be accessed here:



## Figures and Tables

**Table 1:** Demographic characteristics of survey respondents and monthly prevalence of acute, self-reported gastrointestinal illness per category, in British Columbia, Canada, June 2002 to June 2003 (n = 4612).

		**Proportion of Survey Respondents**	**Monthly Prevalence of GI**	
			
			**(%)**	**95% Confidence Interval**
**Gender (n = 4597)**	Male	39.8%	8.5*	(7.3 – 9.9)
	Female	60.2%	10.7*	(9.5 – 11.9)
				
**Age (years) (n = 4309)**	Mean	43.7	-	-
	Median	45	-	-
	0–9	6.8%	17.1*	(12.9 – 21.9)
	10–14	4.2%	15.6*	(10.6 – 21.7)
	15–19	4.1%	8.5	(4.9 – 13.7)
	20–24	4.2%	14.3	(9.6 – 20.2)
	25–64	64.3%	10.4	(9.3 – 11.6)
	65–69	5.3%	7.465	(4.4 – 11.7)
	70–74	4.8%	3.4*	(1.4–6.9)
	75–84	5.2%	4.9*	(2.5 – 8.6)
	85+	1.2%	1.9*	(0.1 – 10.3)
				
**Cultural Group (n = 4536)**	North American	82.2%	10.3*	(9.4 – 11.3)
	European	7.6%	9.3	(6.4 – 12.8)
	African	0.1%	0	-
	Mediterranean	0.6%	7.4	(0.9 – 24.3)
	Asian	7.4%	4.2*	(2.3 – 6.9)
	Native Aboriginal	1.6%	13.7	(6.8 – 23.8)
	South American	0.4%	5.9	(0.2 – 28.7)
	Austral-Asian	0.1%	0	-
				
**Total Household Income (n = 3532)**	<$20,000	15.8%	10.7	(8.3 – 13.6)
	$20,000–<$40,000	24.8%	10.0	(8.1 – 12.2)
	$40,000–<$60,000	24.4%	9.0	(7.2 – 11.2)
	$60,000–<$80,000	15.7%	13.7*	(10.9 – 16.8)
	>$80,000	19.2%	11.1	(8.8 – 13.7)
				
**Highest Level of Education Attained (n = 4536)**	Less than high school	16.17%	8.2	(6.3 – 10.5)
				
	High school diploma	44.4%	9.2	(8.0 – 10.5)
	College or trade diploma	16.6%	10.8	(8.6 – 13.2)
	University, graduate or professional degree	23.0%	11.5*	(9.7 – 13.6)
				
**Number of People in Household**		**Survey Respondents (4612)**	**Cases only (451)**	
	Mean	2.4	2.6	-
	Median	2	2	-
	Minimum	1	1	-
	Maximum	10	8	-

* Prevalence per category level significantly different than all other category levels combined (p < 0.05)

**Table 2:** Odds ratio estimates for variables significantly associated with the prevalence of acute gastrointestinal illness in British Columbia, Canada, June 2002 to June 2003 from a multivariable regression model (total n = 4300 cases n = 444, respondents who did not indicate gender or age were removed from analysis).

**Variable**	**Number of cases**	**Total number of respondents**	**Odds Ratio Estimate**	**95% Confidence Interval**	**P-value**
**Age (years)**					
0–9	50	293	1.8	1.3, 2.5	<0.001
10–14	28	180	1.6	1.1, 2.5	<0.001
15–19	15	176	0.8	0.5, 1.4	0.677
20–24	26	182	1.4	0.9, 2.2	0.003
25–64	289	2763	Reference	Reference	Reference
65–69	17	227	0.7	0.4, 1.2	0.915
70–74	7	205	0.3	0.1, 0.6	0.018
75–84	11	222	0.4	0.2, 0.8	0.108
85+	1	52	0.2	0.0, 1.2	0.099
**Gender**					
Female	290	2609	1.3	1.1, 1.6	0.012
Male	154	1691	Reference	Reference	Reference

**Table 3:** Secondary symptoms and severity of symptoms in respondents reporting acute gastrointestinal illness, in British Columbia, Canada, June 2002 to June 2003.^1^

	**All Cases [451]**	**Both Diarrhoea and Vomiting [114]**	**Diarrhoea Only [286]**	**Vomiting Only [51]**
**Secondary Symptoms **(Number and %)				
Nausea	266 (60%) [440]	92 (84%) [110]	135 (48%) [280]	39 (78%) [50]
Stomach cramps or abdominal pain	331 (75%) [444]	92 (84%) [109]	220 (77%) [284]	19 (37%) [51]
Fever	138 (32%) [437]	52 (47%) [111]	70 (25%) [275]	16 (31%) [51]
Chills	194 (44%) [445]	67 (60%) [111]	110 (39%) [283]	17 (33%) [51]
Muscle or joint pain or stiffness	183 (42%) [439]	53 (48%) [110]	118 (42%) [281]	12 (25%) [48]
Headache	221 (51%) [436]	64 (59%) [109]	136 (49%) [278]	21 (43%) [49]
Excessive Thirst	175 (40%) [443]	54 (49%) [111]	110 (39%) [281]	11 (22%) [51]
Lethargy or extreme tiredness	286 (64%) [445]	93 (82%) [113]	170 (61%) [281]	23 (45%) [51]
Sore throat or runny nose	140 (31%) [447]	38 (33%) [114]	86 (31%) [282]	16 (31%) [51]
Coughing or sneezing	136 (30%) [446]	33 (29%) [113]	89 (31%) [283]	14 (28%) [50]

**Duration **(days)				
Mean, median and range of duration of general illness	3.7, 2, (1–42) [443]	4.5, 3, (1–30) [113]	3.7, 2, (1–42) [279]	1.9, 1, (1–7) [51]
Mean, median and range of duration of vomiting	1.6, 1, (1–25) [164]	1.8, 1, (1–25) [113]	-	1.2, 1, (1–4) [51]
Mean, median and range of duration of diarrhoea	3.2, 2, (<1–42) [392]	3.3, 2, (1–20) [113]	3.2, 2, (<1–42) [279]	-

1. Numbers in square brackets are the total number of respondents per question per category

**Table 4:** Number of telephone calls and visits to health care providers by cases of acute gastrointestinal illness in British Columbia, Canada, June 2002 to June 2003.

**Health Care Provider**	**Number of Calls (n = 62 cases)**	**Number of Visits (n = 52 cases)**
Physician	51	51
Pharmacist	5	0
Nurse practitioner	3	3
Nurse	3	1
Homeopath	1	0

**Table 5:** Type and number of samples requested and submitted by cases of acute, self-reported gastrointestinal, in British Columbia, Canada, June 2002 to June 2003.

**Sample**	**Number of people who were requested to submit**	**Total number of people who submitted samples**
**Urine only**	2	2
**Blood only**	8	6
**Stool only**	3	4
**Urine and Blood**	6	6
**Blood and Stool**	2	0
**Urine and Stool**	0	0
**Urine, Blood and Stool**	7	6

**Total**	**28**	**24**

## References

[B1] Guerrant R, Kosek M, Moore S, Lorntz B, Brantley R, Lima AAM (2002). Magnitude and impact of diarrheal diseases. Arch Med Res.

[B2] Sockett PN (1991). The economic implications of human salmonella infection. J Appl Bacteriol.

[B3] Kosek M, Bern C, Guerrant RL (2003). The global burden of diarrhoeal disease, as estimated from studies published between 1992 and 2000. Bulletin of the World Health Organization.

[B4] Sockett P (1993). Social and economic aspects of food-borne disease. Food Policy.

[B5] Todd ECD (1989). Preliminary estimates of costs of foodborne disease in Canada and costs to reduce Salmonellosis. J Food Prot.

[B6] de Wit MAS, Koopmans MPG, Kortbeek LM, Wannet WJB, Vinjé J, van Leusden F, Bartelds AIM, van Duynhoven YTHP (2001). Sensor, a population-based cohort study on gastroenteritis in the Netherlands, incidence and etiology. Am J Epidemiol.

[B7] Herikstad H, Yang S, Van Gilder TJ, Vugia D, Hadler J, Blake P, Deneen V, Shiferaw B, Angulo FJ (2002). A population-based estimate of the burden of diarrhoeal illness in the United States: FoodNet, 1996–7. Epidemiol Infect.

[B8] Imhoff B, Morse D, Shiferaw B, Hawkins M, Vugia D, Lance-Parker S, Hadler J, Medus C, Kennedy M, Moore MR, Van Gilder T (2004). Burden of self-reported acute diarrhoeal illness in FoodNet surveillance areas, 1998–1999. Clin Infect Dis.

[B9] Scallan E, Fitzgerald M, Collins C, Daly L, Devine M, Igoe D, Quigley T, Robinson T, Smyth B (2004). Acute gastroenteritis in Northern Ireland and the Republic of Ireland: a telephone survey. Commun Dis Public Health.

[B10] Roberts JA, Cumberland P, Sockett PN, Wheeler J, Rodrigues LC, Sethi D, Roderick PJ (2003). The study of infectious intestinal disease in England: socio-economic impact. Epidemiol Infect.

[B11] van den Brandhof WE, De Wit GA, de Wit MA, van Duynhoven YT (2004). Costs of gastroenteritis in The Netherlands. Epidemiol Infect.

[B12] Kuusi M, Aavitsland P, Gondrosen B, Kapperud G (2003). Incidence of gastroenteritis in Norway – a population-based survey. Epidemiol Infect.

[B13] Majowicz SE, Dore K, Flint J, Edge VL, Read S, Buffett MC, McEwen S, McNab WB, Stacey D, Sockett PN, Wilson JB (2004). Magnitude and Distribution of Acute, Self-Reported Gastrointestinal Illness in a Canadian Community. Epidemiol Infect.

[B14] Statistics Canada (2002). 2001 Census. http://www.statcan.ca.

[B15] Health Canada An Introductory Letter in Advance of a Telephone Survey may Increase Response Rate. CCDR.

[B16] Rothman KJ, Greenland S (1998). Modern Epidemiology.

[B17] Wheeler JG, Sethi D, Cowden JM, Wall PG, Rodrigues LC, Tompkins DS, Hudson MJ, Roderick PJ (1999). Study of infectious intestinal disease in England: rates in the community, presenting to general practice, and reported to national surveillance. BMJ.

[B18] Public Health Agency of Canada (2003). Major Chronic Diseases Surveillance Online. http://www.phac-aspc.gc.ca.

[B19] Majowicz SE, Hall G, Scallan E, Adak GK, Gauci C, Jones T, O'Brien S, Henao O, Sockett PN (2006). Development of a common symptom-based case definition for gastroenteritis: An international analysis [abstract]. Proceedings of the International Conference on Emerging Infectious Diseases 2006.

[B20] Majowicz S, Stacey D (2005). The use of clustering to analyze symptom-based case definitions for acute gastrointestinal illness [abstract]. Proceedings of the International Joint Conference on Neural Networks 2005.

[B21] Palmer S, Houston H, Lervy B, Ribeiro D, Thomas P (1996). Problems in the diagnosis of foodborne infection in general practice. Epidemiol Infect.

[B22] Hoogenboom-Verdegaal AMM, De Jong JC, During M, Hoogenveen R, Hoekstra JA (1994). Community-based study of the incidence of gastrointestinal diseases in the Netherlands. Epidemiol Infect.

[B23] Frühwirth M, Karmaus W, Moll-Schüler I, Brösl S, Mutz I (2001). A prospective evaluation of community acquired gastroenteritis in paediatric practices: impact and disease burden of rotavirus infection. Arch Dis Child.

[B24] Food Standards Agency (2000). A report of the study of infectious intestinal disease in England.

[B25] Monto AS, Koopman JS (1980). The Tecumseh Study. Am J Epidemiol.

[B26] Kagan LJ, Aiello AE, Larson E (2002). The role of the home environment in the transmission of infectious diseases. J Community Health.

[B27] Odoi A, Martin SW, Michel P, Holt J, Middleton D, Wilson J (2003). Geographical and temporal distribution of human giardiasis in Ontario, Canada. Int J Health Geogr.

[B28] Michel P, Wilson JB, Martin SW, Clarke RC, McEwen SA, Gyles CL (1999). Temporal and geographical distributions of reported cases of *Escherichia coli *O157:H7 infection in Ontario. Epidemiol Infect.

[B29] Mounts AW, Ando T, Koopmans M, Bresee JS, Noel J, Glass RI (2000). Cold weather seasonality of gastroenteritis associated with Norwalk-like viruses. J Infect Dis.

[B30] Cook SM, Glass RI, LeBaron CW, Ho MS (1990). Global seasonality of rotavirus infections. Bulletin of the World Health Organization.

[B31] Olesen B, Neimann J, Bottiger B, Ethelberg S, Schiellerup P, Jensen C (2005). Etiology of diarrhea in young children in Denmark: a case-control study. J Clin Microbiol.

[B32] Statistics Canada (2002). 2001 Residential Telephone Survey. http://www.statcan.ca.

